# Induction Regimens in High‐Risk Neuroblastoma: Systematic Review of Response Rates and Toxicities

**DOI:** 10.1002/cam4.71312

**Published:** 2025-11-23

**Authors:** Samantha D. Martin, Eva C. Robinson, Edie Weller, Rochelle Bagatell, Lucas Moreno, Steven G. DuBois

**Affiliations:** ^1^ Department of Pediatrics, Dana‐Farber/Boston Children's Cancer and Blood Disorders Center Harvard Medical School Boston Massachusetts USA; ^2^ Boston Children's Hospital Biostatistics and Research Design Center (BARD) Boston Massachusetts USA; ^3^ Department of Pediatrics Children's Hospital of Philadelphia and Perelman School of Medicine Philadelphia Pennsylvania USA; ^4^ Department of Pediatric Oncology Vall d'Hebron Hospital Barcelona Spain

## Abstract

**Background:**

Numerous induction therapies have been evaluated for high‐risk neuroblastoma (HRNBL). It is not known how these regimens' response rates nor toxicities compare. We aimed to describe and compare key features of HRNBL induction regimens and their associations with study‐level end‐induction response (EIR).

**Methods:**

We performed a systematic review (PubMed) of prospective trials of frontline HRNBL therapy published January 1, 1995–October 31, 2024 that reported EIR. EIR was measured as partial response or better (PR+) per protocol response criteria.

**Results:**

1395 unique titles were screened, yielding 95 abstracts. Of these, 29 publications evaluating 36 induction regimens met inclusion criteria, with a median of 64.5 patients (range: 7–652) per regimen. Median cycle number was 6 (range: 2–9), cycle length was 21 days (10–28), and total duration of induction was 18 weeks (11.4–36). An alkylator and a platinum agent were used in all regimens. Only six regimens (16.7%) included a novel agent. The median study level EIR rate (PR+) was 84.4% (64.3–100), with a weighted average by the number of participants of 79.4%. Study level EIR did not vary over time. Anthracycline‐containing regimens had higher EIRs. Dose cisplatin intensity was negatively associated with EIR. The median toxic death rate was 0% (0–4.1).

**Conclusions:**

Over the past 30 years, induction regimens have relied heavily on conventional chemotherapy. Despite differences in agents, doses, and duration, study‐level EIR rates have not improved over time. Future induction regimens incorporating novel agents will be crucial to improve EIR and reduce toxicities.

## 1 Introduction

Neuroblastoma is the most common cancer of infants, and the most common extracranial solid tumor in childhood. Patients are classified as having low‐, intermediate‐, or high‐risk disease using clinical, histological, and molecular features [1, 2, 3]. Despite maximally intensive multi‐modal therapies, cure rates for patients with high‐risk disease remain significantly lower than those of other risk groups, with a 3‐year event‐free survival (EFS) rate of 51.1% from the time of diagnosis for patients enrolled in the Children's Oncology Group (COG) trial (ANBL0532) and a similar 3‐year EFS rate of 44.0% using the rapid COJEC regimen in a recent International Society of Pediatric Oncology European Neuroblastoma (SIOPEN) group HR‐NBL trial [4, 5].

Therapy for patients with high‐risk neuroblastoma begins with multiagent induction chemotherapy. Distinct induction regimens are in use internationally, which vary by agents employed, duration, cycle number, and dose intensity (Appendix S1) [4, 5, 6, 7, 8, 9, 10, 11]. However, few randomized controlled trials comparing induction regimens have been conducted, leading to a lack of data regarding comparative efficacy and toxicity [5, 12, 13, 14, 15]. Moreover, whether specific features of induction regimens are associated with differential study‐level end‐induction response (EIR) rates or toxicity is not known. To investigate these potential associations and to inform future clinical trials, we performed a systematic review to (i) describe and compare key features of induction regimens for high‐risk neuroblastoma, (ii) describe the study‐level EIR rates for high‐risk neuroblastoma induction regimens and compare based upon key features of these regimens, and (iii) report the study‐level incidence of common hematologic and non‐hematologic toxicities.

## 2 Materials and Methods

We performed a systematic literature review of prospective clinical trials of upfront induction therapy for high‐risk neuroblastoma (PROSPERO ID: CRD42024608782) in alignment with the Preferred Reporting Items for Systematic Reviews and Meta‐Analyses (PRISMA) guidelines [16]. Covidence software (Veritas Health Innovation, Melbourne, Australia) was used for citation management and data extraction.

### 2.1 Search Strategy and Selection Criteria

Eligible publications were primary manuscripts describing a pilot, phase II, or phase III clinical trial of frontline therapy for patients with high‐risk neuroblastoma that reported the response rate to induction therapy. Case reports, case series, publications reported in a language other than English, publications without available full text, secondary or long‐term follow‐up of trials otherwise included in the analysis, publications describing relapsed/refractory or non‐high‐risk, and retrospective analyses were excluded.

The electronic database PubMed was utilized in addition to reference checking to identify potentially eligible publications. A complete list of search terms utilized to compile potentially eligible publications is provided (Appendix S2). Only articles published between January 1, 1995 and October 31, 2024 were included. Titles were first screened by a single reviewer, abstracts were then screened by two reviewers, and the full text was reviewed by a single reviewer to confirm final eligibility. If disagreement about the inclusion of a publication was noted at the abstract stage, a third reviewer was available to adjudicate, but this step was not ultimately needed.

### 2.2 Variable Definitions

Collected data from the original publications followed definitions used by the original authors, without reclassification. Induction length was defined as the number of planned weeks over which induction therapy was scheduled to be delivered. The number of chemotherapy cycles was defined as the number of planned induction chemotherapy cycles. The cumulative chemotherapy doses were defined as the total planned induction dose of each agent in mg/m^2^ and cyclophosphamide equivalent dose (CED) was calculated [17]. Dose intensities were calculated by the total planned dose during induction of the drug in mg/m^2^ divided by the number of weeks over which the induction regimen was scheduled, to give an aggregate dose intensity in mg/m^2^/week [18, 19]. Regimens without reported or calculable planned duration of induction were excluded from the calculation of planned dose intensities.

All endpoints used in the analysis are at the study level. The primary endpoint for this analysis was EIR rate, defined as the percent of evaluable participants with a partial response or better (PR, VGPR, or CR) at end‐induction using the International Neuroblastoma Response Criteria (INRC) version in use at the time of study publication. End‐induction complete response (CR), end‐induction progressive disease (PD), EFS, and overall survival (OS) rates were also collected, if reported.

### 2.3 Statistical Analysis

Weighted average response rates were calculated using the number of patients treated for each regimen relative to the total number of patients for all regimens combined. Binomial exact 95% confidence intervals were calculated for each study‐level EIR, CR, and PD rate based on the number of participants. Weighted univariate linear regression was used to evaluate the association between dependent variables (study‐level EIR, CR, PD rates as well as 5‐ and 3‐year EFS and OS proportions) and independent variables. Box and whisker plots were used to graphically display the data. In these plots, the median is represented by the line in the middle of the box, and the interquartile range is represented by the box. Statistically significant associations were those with *p* < 0.05, and marginal associations were those with *p* < 0.1 but ≥ 0.05. Statistical software R (R Core Team, 2021; Vienna, Austria) was utilized for data analysis and visualization.

## 3 Results

### 3.1 Key Characteristics of Eligible Induction Regimens

**FIGURE 1:**
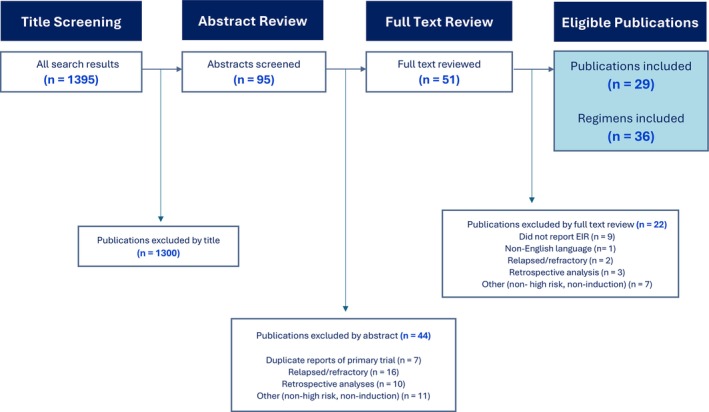
PRISMA diagram displaying systematic review and screening process.

The PubMed search yielded 1395 unique publications, from which 95 abstracts were screened. Of these, 29 publications evaluating 36 induction regimens met inclusion criteria (Figure 1).

Key characteristics of the eligible regimens are shown (Table 1). Studies were published between 1995 and 2024, and the earliest accrual began in 1985. The included regimens were administered to a median of 64.5 patients (range: 7–652) per regimen. The median number of planned chemotherapy cycles was 6 (range: 2–9), the median planned cycle length was 21 days (range: 10–28), and the median planned duration of induction was 18 weeks (range: 11.4–36). The median value for the reported median age of included participants was 3.1 years (range: 2–5.3), and the reported median rate of tumor *MYCN* amplification was 43% (range: 10–100, including one trial that only included patients with *MYCN* amplified tumors). Twenty‐four regimens were reported as part of a non‐randomized experimental clinical trial, and 12 were reported as part of a randomized clinical trial. Only eight (22.2%) trials included a randomized induction question. Twenty‐eight (77.8%) regimens were reported as part of a multi‐center trial, and 8 (22.2%) were reported as part of a single‐center trial. Surgical resection was performed after induction therapy in 22 (61.1%) of regimens and during induction therapy in 14 (38.9%) of regimens. All trials reported using the 1993 version of the INRC [20].

**TABLE 1:** Key characteristics of included regimens.

Patient characteristics	*Median of Reported Values (range)*
Median age of participants	3.1 (2–5.3)
Proportion of patients with *MYCN* amplified tumors, of known^a^	43 (10–100)
Regimen characteristics	*Median (range)*
Number of patients treated	64.5 (7–652)
Number of planned chemotherapy cycles	6 (2–9)
Cycle length (days)	21 (10–28)
Induction duration (weeks)	18 (11.4–36)
Cyclophosphamide equivalent dose (CED; mg/m^2^)^b^	8200 (420–16,800)
Cisplatin planned dose intensity (mg/m^2^/week)	22.2 (5–33.3)
Carboplatin planned dose intensity (mg/m^2^/week)	100 (62.5–233.3)
CED planned dose intensity (mg/m^2^/week)	434.1 (28.9–933.3)
Doxorubicin planned dose intensity (mg/m^2^/week)	5.0 (2.8–16.7)
	*Number of Regimens (%)*
Alkylators utilized	36 (100)
Cisplatin utilized	31 (86.1%)
Carboplatin utilized	14 (38.9%)
Any platinum agent utilized	36 (100%)
Anthracycline utilized	27 (75.0%)
Trial characteristics	*Proportion of Known (n)*
Non‐randomized^c^ clinical trial Randomized^c^ clinical trial	66.7% (24) 33.3% (12)
Randomized induction question	22.2% (8)
Multi‐center trial Single center trial	77.8% (28) 22.2% (8)
Multiple induction regimens included Single induction regimen included	36.1% (13) 63.9% (23)
Surgical resection after induction Surgical resection during induction	61.1% (22) 38.9% (14)
COG^d^ and legacy groups SIOPEN^e^ GPOH^f^ JCCG^g^ Other or not affiliated with cooperative group	22.2% (8) 11.1% (4) 8.3% (3) 8.3% (3) 50% (18)
Accrual started 1985–1989 Accrual started 1990–1994 Accrual started 1995–1999 Accrual started 2000–2004 Accrual started 2005–2009 Accrual started 2010–2014 Accrual started 2015–2019	2 (5.9%)^h^ 0 (0%) 14 (41.2%) 7 (20.6%) 2 (5.9%) 6 (17.6%) 3 (8.8%)
Regimen published 1995–1999 Regimen published 2000–2004 Regimen published 2005–2009 Regimen published 2010–2014 Regimen published 2015–2019 Regimen published 2020–2024	4 (11.1%) 6 (16.7%) 6 (16.7%) 6 (16.7%) 3 (8.3%) 11 (30.6%)

^a^ *MYCN* amplification rate reported in 80.6% of included regimens.

^b^ Excluding regimens that did not utilize alkylators.

^c^ Trials were considered randomized if there was any randomization event. Participants were not necessarily randomized to an induction regimen specifically.

^d^ Children's Oncology Group.

^e^ International Society of Pediatric Oncology Europe Neuroblastoma Group.

^f^ German Society for Pediatric Oncology and Hematology.

^g^ Japan Children's Cancer Group.

^h^ Year accrual started was not reported for two regimens. Calculated proportions are out of the 34 with known accrual start year.

**FIGURE 2:**
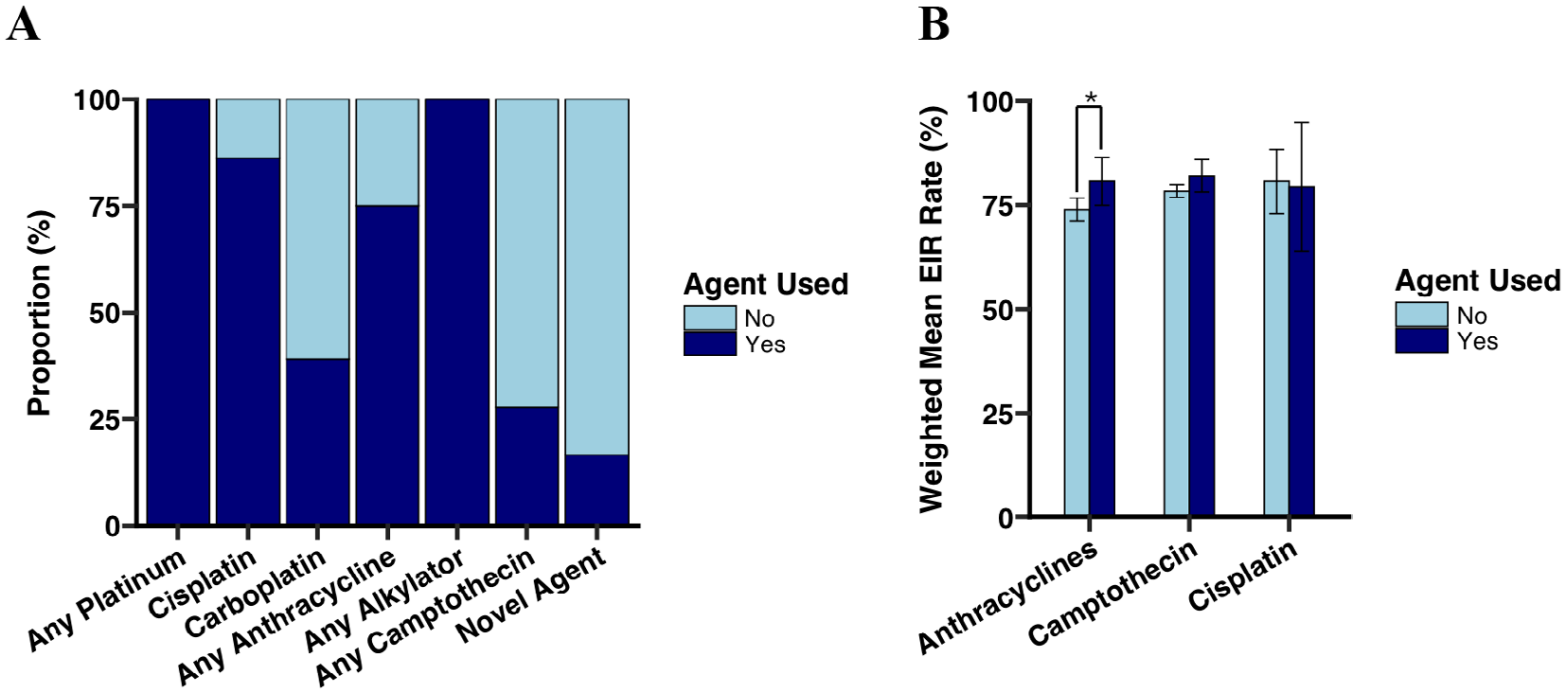
Chemotherapy agent utilization and impact on study‐level end induction rate (EIR). (A) Proportion of regimens using each chemotherapy agent; (B) Weighted mean EIR rate by use of anthracycline (left; *p* = 0.036 between weighted group means), camptothecin (middle; *p* = 0.203), and cisplatin (right; *p* = 0.866). * indicates statistical significance (*p* < 0.05).

Figure 2A displays the proportion of regimens using each chemotherapy agent. An alkylator and a platinum agent were used in all 36 regimens. Cisplatin was used in 31 (86.1%), carboplatin in 14 (38.9%), vincristine in 26 (72.2%), and anthracyclines in 27 (75%). Ten regimens (27.9%) included at least one camptothecin. Only six regimens (16.7%) included a novel agent, including MIBG, arsenic trioxide, anti‐GD2 antibodies, and difluoromethylornithine.

### 3.2 End Induction Response Rates by Regimen

**FIGURE 3:**
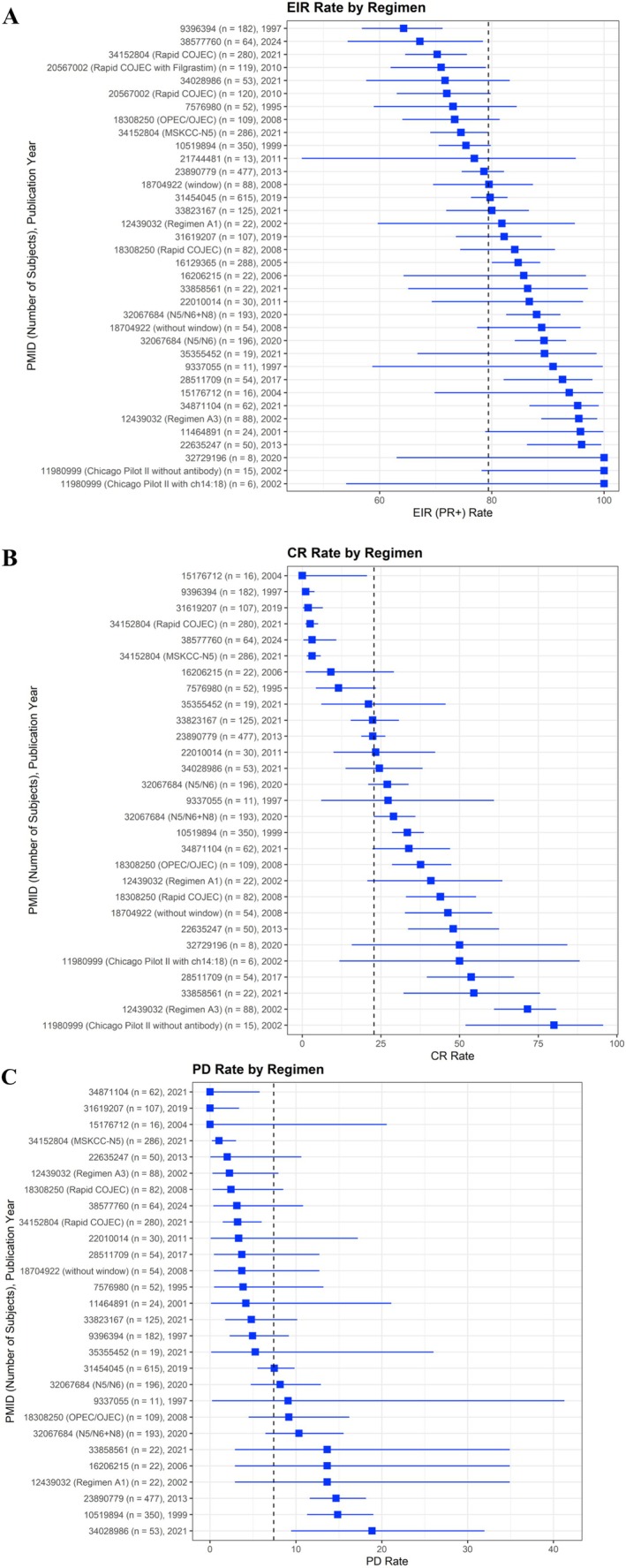
Summary of study‐level (A) EIR, (B) CR, and (C) PD by regimen PMID, with trial arm in parentheses if multiple induction regimens tested. Dashed lines represent weighted average rates by number of participants (EIR 79.4%, CR 22.8%, and PD 7.4%). Regimens are sorted by outcome rate, with 95% binomial exact confidence intervals displayed.

**FIGURE 4:**
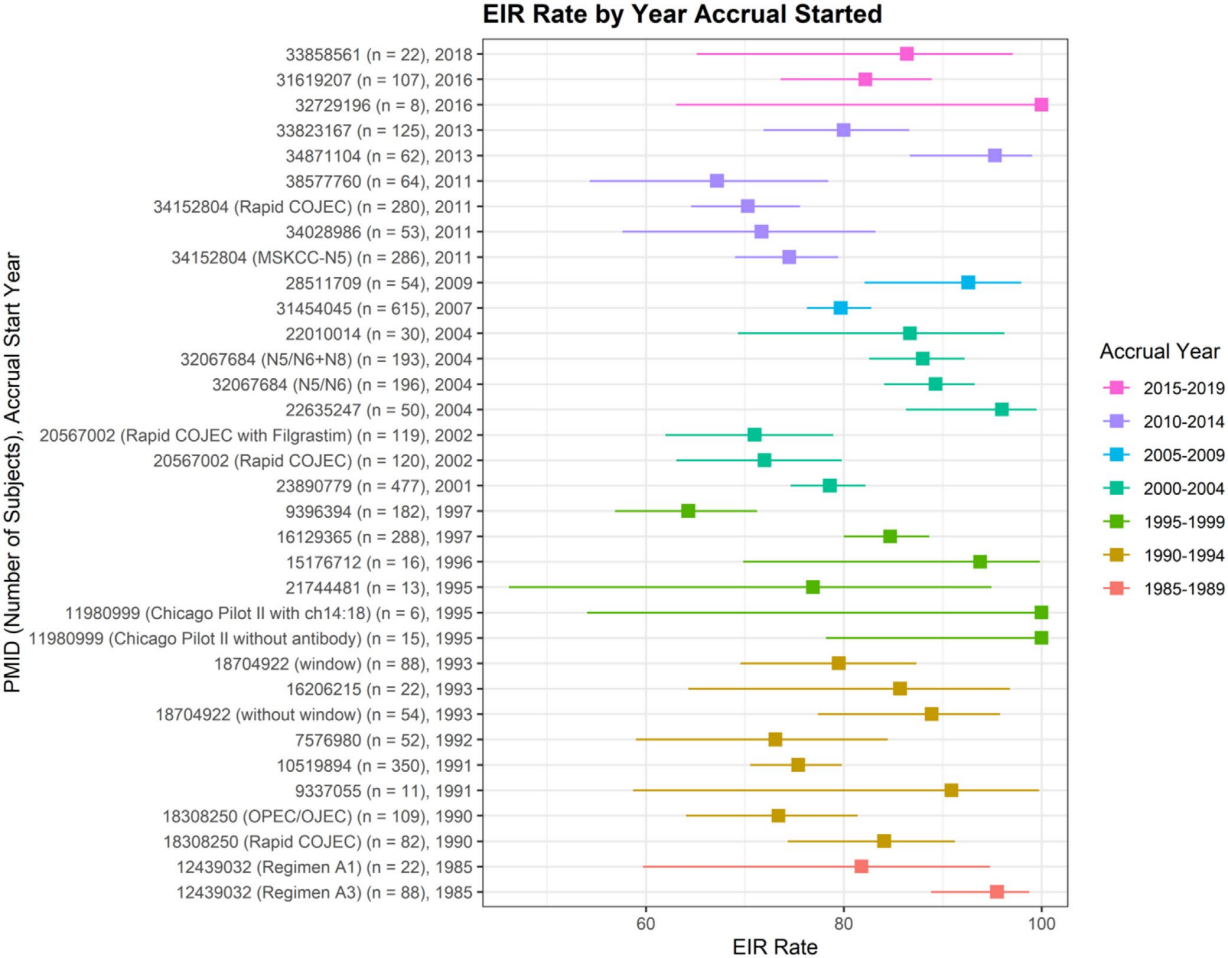
Study‐level end induction response (EIR) rate with 95% exact binomial confidence intervals by year accrual started, bucketed in 5‐year intervals from 1985–1989 (*n* = 2; red), 1990–1994 (*n* = 8; gold), 1995–1999 (*n* = 6; dark green), 2000–2004 (*n* = 7; teal), 2005–2009 (*n* = 2; blue), 2010–2014 (*n* = 6; purple), and 2015–2019 (*n* = 3; pink).

Study level EIR rates of included regimens are displayed in Figure 3A and in Appendix S3. The median end induction response rate (EIR) of partial response or better (PR+) across 36 regimens was 84.4% (range: 64.3–100), with a weighted average by number of participants of 79.4%. Multicenter trials had lower EIR compared to single center trials, with a weighted mean EIR of 78.5% versus 95.3%, respectively (*p* = 0.002). Regimens with scheduled surgery during induction were marginally associated with a higher weighted mean EIR compared to those with surgery scheduled after completion of induction (83.1% versus 78.0%, respectively; *p* = 0.092). Excluding the 6 regimens that evaluated a novel agent, the weighted average EIR was 79.2% for the remaining 30 regimens that utilized only conventional chemotherapy during induction. There was not a significant trend over time in study level EIR by accrual start year (*p* = 0.352; Figure 4).

The median CR rate across 28 regimens was 29.6% (range: 0–80; reported in 77.8%; Figure 3B), with a weighted average by number of participants of 22.8%. The median PD rate across 26 regimens was 4.6% (range: 0–18.9; reported in 72.2% of regimens; Figure 3C), with a weighted average by number of participants of 7.4%. Metastatic EIR was only reported for four regimens and therefore not further analyzed.

Univariate weighted linear regression models were used to examine the association of several regimen characteristics with study‐level EIR, CR, and PD rates (Table 2). Higher cisplatin dose intensity (in mg/m^2^/week) was significantly associated with lower mean EIR rate (coefficient: −0.364 [−0.707, −0.021]; *p* = 0.038), though study‐level EIR for regimens that used cisplatin did not differ significantly compared with those that did not use cisplatin (cisplatin weighted group mean 79.4%; no cisplatin weighted group mean 80.7%; *p* = 0.866; Figure 2B). There was no association between cumulative cisplatin dose and EIR. Anthracycline‐containing regimens had a statistically significantly higher study‐level mean EIR (anthracycline weighted group mean 80.7%; no anthracycline weighted group mean 73.9%; *p* = 0.036; Figure 2B). No difference was observed in weighted mean EIR by use of camptothecin (camptothecin weighted group mean 82.1%; no camptothecin weighted group mean 78.4%; *p* = 0.203; Figure 2B). No other evaluated features of induction regimens were significantly associated with EIR (Table 2).

**TABLE 2:** EIR, CR, and PD rates and weighted linear regression coefficients and weighted correlation. Bold denotes *p* < 0.05, and italics denote marginal associations (*p* < 0.1).

Variable	EIR			CR			PD		
	Correlation coefficient adjusted for weighting	Model coefficient (95% CI)	*p*	Correlation coefficient adjusted for weighting	Model coefficient (95% CI)	*p*	Correlation coefficient adjusted for weighting	Model coefficient (95% CI)	*p*
Induction length (weeks)	0.229	0.384 [−0.273, 1.04]	0.240	**0.583**	**1.949 [0.716, 3.183]**	**0.004**	0.312	0.415 [−0.192, 1.023]	0.169
Cycle length (days)	0.192	0.299 [−0.316, 0.914]	0.327	*0.386*	*1.103 [−0.092, 2.299]*	*0.069*	*0.390*	*0.441 [−0.059, 0.941]*	*0.080*
Number of cycles	0.118	0.674 [−1.312, 2.661]	0.495	0.253	3.348 [−1.809, 8.506]	0.194	−0.049	−0.214 [−2.038, 1.609]	0.810
Cumulative cyclophosphamide equivalent dose (CED; mg/m^2^)	0.095	0 [0, 0.001]	0.580	*0.355*	*0.001 [0, 0.002]*	*0.064*	**0.459**	**0.0004 [0, 0.001]**	**0.018**
Cumulative cisplatin dose (mg/m^2^)	−0.182	−0.011 [−0.031, 0.009]	0.289	0.034	0.004 [−0.048, 0.057]	0.863	−0.151	−0.006 [−0.023, 0.011]	0.460
Cumulative carboplatin dose (mg/m^2^)	−0.112	−0.001 [−0.004, 0.002]	0.522	−0.030	−0.001 [−0.008, 0.007]	0.883	*−0.355*	*−0.002 [−0.004, 0]*	*0.082*
Cumulative doxorubicin dose (mg/m^2^)	0.263	0.021 [−0.006, 0.049]	0.121	0.120	0.02 [−0.048, 0.089]	0.544	**0.447**	**0.025 [0.004, 0.046]**	**0.022**
Dose intensity of CED (mg/m^2^/week)	−0.023	−0.001 [−0.01, 0.009]	0.906	0.088	0.004 [−0.018, 0.026]	0.689	**0.439**	**0.008 [0, 0.016]**	**0.047**
Dose intensity of cisplatin (mg/m^2^/week)	**−0.394**	**−0.364 [−0.707, −0.021]**	**0.038**	**−0.481**	**−0.866 [−1.582, −0.15]**	**0.020**	−0.342	−0.244 [−0.565, 0.078]	0.129
Dose intensity of carboplatin (mg/m^2^/week)	−0.186	−0.025 [−0.081, 0.03]	0.354	−0.211	−0.052 [−0.165, 0.061]	0.345	*−0.422*	*−0.041 [−0.085, 0.003]*	*0.064*
Dose intensity of doxorubicin (mg/m^2^/week)	0.115	0.143 [−0.354, 0.641]	0.559	−0.103	−0.254 [−1.366, 0.857]	0.639	*0.391*	*0.382 [−0.049, 0.814]*	*0.079*

Longer total induction length was significantly associated with a higher mean CR rate (coefficient: 1.949 [0.716, 3.183]; *p* = 0.004). Higher cisplatin dose intensity was significantly associated with a lower mean CR rate (coefficient: −0.866 [−1.582, −0.15]; *p* = 0.02). Cumulative CED, dose intensity of CED, and cumulative doxorubicin dose were each significantly associated with an increase in mean PD rate (coefficient: 0.0004 [0, 0.001], *p* = 0.018; coefficient: 0.025 [0.004, 0.046], *p* = 0.047; and coefficient: 0.025 [0.004, 0.046], *p* = 0.022 respectively).

### 3.3 EFS and Overall Survival Endpoints

EFS at 3 or 5 years was reported in *n* = 17 (47.2%) of regimens, and OS at 3 or 5 years was reported in *n* = 16 (44.4%). Half of the included regimens did not report EFS nor OS. Median reported EFS at 3 and 5 years was 37.8% (range: 24.2–73.7) and 37.0% (18.2–71.4), respectively. Median reported OS at 3 and 5 years was 62.5% (range: 48–86) and 48% (21.2–77).

End induction response (EIR) rate for a given regimen was marginally associated with an increase of 5‐year EFS (coefficient 1.093 [−0.191, 2.378]; *p* = 0.082; Appendix S4), though it is acknowledged that post‐induction therapies could impact overall survival and EFS outcomes. However, there was no significant association of EIR rate with 3‐year, 5‐year OS nor 3‐year EFS. Small numbers of studies in each year category precluded reliable statistical analysis of trends over time in EFS and OS.

### 3.4 Toxicity Endpoints

Key toxicities are displayed in Appendix S5. Rates of toxic death during induction were reported for 33 regimens (91.7% of regimens), with 12 of 33 (36%) regimens having at least 1 toxic death. The median toxic death rate during induction was 0% (range: 0–4.1). The median documented infection rate was 41.3% (range: 25–100, reported in 19.4%) and the median rate for completing induction therapy was 93.0% (range: 66.7–100, reported in 61.1%). The incidence of serious adverse events (SAEs) in induction was not reported in any publication. The rate of treatment delays due to AE during induction was only reported in 5.6% of regimens, with a median reported incidence of 36.5% (range: 35–38).

Other induction toxicities were infrequently reported, and, when reported, were inconsistently defined between publications (see Appendix S5 for details). For example, data on the frequency of ototoxicity and secondary malignancies were only available for 11.1% and 5.6% of regimens, respectively.

## 4 Discussion and Conclusions

Despite wide variation in induction regimens, approximately 80% of patients will have at least a partial response at the end of induction for upfront high‐risk neuroblastoma therapy. This outcome has not meaningfully improved over time. There remains a strong reliance on cytotoxic chemotherapy, with only 16.7% of induction regimens utilizing a novel agent.

To inform future induction strategies in this disease, we evaluated potential regimen features that might be associated with differential responses. Study‐level EIRs were higher with anthracycline‐containing regimens, though higher cumulative doxorubicin doses were also associated with higher rates of PD. The specific reason for this finding is unclear, and future mechanistic studies will be needed to evaluate this further. Many contemporary regimens used by GPOH, COG, and MSKCC include anthracyclines in their induction regimens. Rapid COJEC does not contain anthracyclines, and the most recently reported phase III trial of rapid COJEC demonstrated an EIR of 70.3% compared to our observed weighted average of 79.4%. However, confounding this comparison is the difference in surgical timing, which impacted EIR assessments. However, the use of anthracyclines is not without its cost in acute and late effects. A dose‐dependent effect has been observed between total anthracycline exposure and the risk of heart failure [21]. Our data could provide evidence that future induction regimens should include some exposure to anthracyclines as a drug class that may be non‐cross resistant with other drug classes commonly utilized while limiting cumulative doses to those less likely to lead to significant late effects.

Our data do not support the observed heavy reliance on cisplatin in induction regimens. Specifically, the inclusion of cisplatin in induction regimens was not associated with EIR, and the dose intensity of cisplatin was negatively associated with both EIR and CR rates. Given the known late effects associated with cisplatin [22, 23, 24, 25, 26, 27, 28], these findings may support the evaluation of other agents during induction. At least one institutional study is currently evaluating the use of carboplatin instead of cisplatin (NCT06528496). Finally, increased induction duration was positively associated with the CR rate, which is contrary to contemporary strategies to reduce the duration of induction [9, 29] and may merit consideration of lower‐intensity regimens given for more cycles in future studies. While longer induction regimens may have higher response rates, high‐risk therapy continues with consolidation and post‐consolidation therapies. Thus, it remains unknown which strategy will result in improved overall survival and reduced long‐term toxicities.

Induction‐specific toxicity data were inconsistently defined and infrequently reported; thus, conclusions about comparative toxicities were limited. Short‐ and long‐term toxicities of pediatric oncology chemotherapy regimens are considerable and have an important impact on patients' quality of life [5, 30, 31, 32, 33, 34, 35, 36, 37]. Major efforts should be undertaken to improve the consistent reporting of induction‐level toxicities.

The observed lack of appreciable improvement in EIR over the last 30 years is sobering. Given the intensity of the treatment regimens reported in our analysis, it seems unlikely that further dose intensification of conventional chemotherapy will be feasible or effective. Instead, the field appears to have reached a ceiling effect with chemotherapy alone, and integration of novel agents may be needed to further improve EIR. For example, there has been tremendous interest in the combination of chemotherapy with anti‐GD2 monoclonal antibody (referred to as “chemoimmunotherapy”) based upon activity in children with relapsed neuroblastoma [38, 39, 40]. Additional novel agents being evaluated in induction therapy include lorlatinib for *ALK*‐driven tumors [41] and novel radiotherapeutics [42]. It is important to note that although EIR has not improved over time, long‐term outcomes have improved over time due to innovations in the consolidation and post‐consolidation phases of therapy [43].

Strengths of this study include the systematic approach that yielded a wide variety of included induction regimens, representing multiple cooperative groups and regimens from around the globe across multiple decades. However, this study is limited by several important factors. First, all data captured are based on study‐level data, thus all chemotherapy doses, intensities, and induction cycle number and length are as planned rather than as received. As this analysis is not based on patient‐level data, confounders that might impact EIR, such as *MYCN* and 11q status [44] could not be used as potential covariates. Furthermore, overall and metastatic response criteria and imaging sensitivity evolved over the included time period [20, 45, 46], and it was not feasible to re‐classify patients according to a contemporary standard. Last, the lack of uniform toxicity reporting significantly limits conclusions from this portion of the analysis.

Over the past three decades, induction regimens have relied heavily on conventional chemotherapy and may have reached a ceiling effect. Despite differences in agents, doses, and duration, study level EIR rates have not improved over time. Future induction regimens should incorporate targeted agents and immunotherapies to improve EIR and reduce toxicities.

## Author Contributions

**Samantha D. Martin:** formal analysis, visualization, writing – original draft, data curation, investigation. **Eva C. Robinson:** formal analysis, visualization. **Edie Weller:** visualization, formal analysis. **Rochelle Bagatell:** conceptualization, writing – review and editing. **Lucas Moreno:** conceptualization, writing – review and editing. **Steven G. DuBois:** conceptualization, investigation, writing – review and editing, supervision.

## Conflicts of Interest

SGD reports consulting fees from EMD Serono, InhibRx, and Merck.

## Supporting information


**Appendix S1:** Regimen characteristics of most commonly used European and North American approaches to frontline treatment of patients with high‐risk neuroblastoma.
**Appendix S2:** Search terms utilized for initial reference list.
**Appendix S3:** Regimens from included studies with number of patients treated, study level end induction response rate (EIR), complete response rate (CR), and progressive disease (PD) rate.
**Appendix S4:** Study level end induction response rate (EIR) as a predictor of 3‐year EFS, 5‐year EFS, 3‐year OS, and 5‐year OS using linear regression weighted by number of subjects.
**Appendix S5:** Key toxicities of included regimens.

## Data Availability

The data that supports the findings of this study are available in the Supporting Information of this article.
