# Supplementary material for: Induction Regimens in High‐Risk Neuroblastoma: Systematic Review of Response Rates and Toxicities
Source: Cancer Med. 2025 Nov 23;14(22):e71312. doi: 10.1002/cam4.71312 (PMC12640792; doi:10.1002/cam4.71312)
Supplement: Supplementary file 1 — Appendix S1: Regimen characteristics of most commonly used European and North American approaches to frontline treatment of patients with high‐risk neuroblastoma. Appendix S2: Search terms utilized for initial reference list. Appendix S3: Regimens from included studies with number of patients treated, study level end induction response rate (EIR), complete response rate (CR), and progressive disease (PD) rate. Appendix S4: Study level end induction response rate (EIR) as a predictor of 3‐year EFS, 5‐year EFS, 3‐year OS, and 5‐year OS using linear regression weighted by number of subjects. Appendix S5: Key toxicities of included regimens. [file CAM4-14-e71312-s001.docx]

Appendix S1: Regimen characteristics of most commonly used European and North American approaches to frontline treatment of patients with high-risk neuroblastoma

| **Regimen** | **Agents used and doses by cycle** |
| --- | --- |
| Rapid COJEC^7^ | Cycle 1: Vincristine 1.5 mg/m^2^/dose on Day 1, Carboplatin 750 mg/m^2^/dose on Day 1, Etoposide 175 mg/m^2^/dose on Days 1-2  Cycle 2: Vincristine 1.5 mg/m^2^/dose on Day 1, Cisplatin 80mg/m2/dose on Day 1  Cycle 3: Vincristine 1.5 mg/m^2^/dose on Day 1, Etoposide 175 mg/m^2^/dose on Days 1-2, Cyclophosphamide 1,050 mg/m^2^/dose on Days 1-2  Cycle 4: Vincristine 1.5 mg/m^2^/dose on Day 1, Cisplatin 80mg/m2/dose on Day 1  Cycle 5: Vincristine 1.5 mg/m^2^/dose on Day 1, Carboplatin 750 mg/m^2^/dose on Day 1, Etoposide 175 mg/m^2^/dose on Days 1-2  Cycle 6: Vincristine 1.5 mg/m^2^/dose on Day 1, Cisplatin 80mg/m2/dose on Day 1  Cycle 7: Vincristine 1.5 mg/m^2^/dose on Day 1, Etoposide 175 mg/m^2^/dose on Days 1-2, Cyclophosphamide 1,050 mg/m^2^/dose on Days 1-2  Cycle 8: Vincristine 1.5 mg/m^2^/dose on Day 1, Cisplatin 80mg/m2/dose on Day 1 |
| Memorial Sloan Kettering Cancer Center (MSKCC) N5^8-10^ | Cycle 1: Cyclophosphamide 70 mg/kg/dose on Days 1-2, Doxorubicin 75 mg/m^2^/dose on Day 1, Vincristine 0.067 mg/m^2^/dose on Day 1  Cycle 2: Cyclophosphamide 70 mg/kg/dose on Days 1-2, Doxorubicin 75 mg/m^2^/dose on Day 1, Vincristine 0.067mg/m^2^/dose on Day 1  Cycle 3: Cisplatin 50 mg/m2/dose on Days 1-4, Etoposide 200 mg/m^2^/dose on Days 1-3  Cycle 4: Cyclophosphamide 70 mg/kg/dose on Days 1-2, Doxorubicin 75 mg/m^2^/dose on Day 1, Vincristine 0.067 mg/m^2^/dose on Day 1  Cycle 5: Cisplatin 50mg/m2/dose on Days 1-4, Etoposide 200 mg/m^2^/dose on Days 1-3 |
| Children’s Oncology Group (COG)^4^ | Cycle 1: Cyclophosphamide 400 mg/m^2^/dose on Days 1-5, Topotecan 1.2 mg/m^2^/dose on Days 1-5  Cycle 2: Cyclophosphamide 400 mg/m^2^/dose on Days 1-5, Topotecan 1.2 mg/m^2^/dose on Days 1-5  Cycle 3: Cisplatin 60 mg/m^2^/dose on Days 1-3, Etoposide 200 mg/m^2^/dose on Days 1-3  Cycle 4: Vincristine 2 mg/m^2^/dose on Day 1, Doxorubicin 37.5 mg/m^2^/dose on Days 1-2, Cyclophosphamide 2000 mg/m^2^/dose on Days 1-2  Cycle 5: Cisplatin 50 mg/m^2^/dose on Days 1-4, Etoposide 200 mg/m^2^/dose on Days 1-3  Cycle 6: Vincristine 2 mg/m^2^/dose on Day 1, Doxorubicin 37.5 mg/m^2^/dose on Days 1-2, Cyclophosphamide 2000 mg/m^2^/dose on Days 1-2 |
| German Society for Paediatric Oncology and Haematology (GPOH)^13^ | Cycle 1: Cisplatin 40 mg/m^2^/dose on Days 1-4, Etoposide 100 mg/m^2^/dose on days 1-4, Vindesine 3 mg/m^2^/dose on Day 1  Cycle 2: Vincristine 1.5 mg/m^2^/dose on Days 1 and 8, Dacarbazine 200 mg/m^2^/dose on Days 1-5, Doxorubicin 30 mg/m^2^/dose on Days 6-7  Cycle 3: Cisplatin 40 mg/m^2^/dose on Days 1-4, Etoposide 100 mg/m^2^/dose on days 1-4, Vindesine 3 mg/m^2^/dose on Day 1  Cycle 4: Vincristine 1.5 mg/m^2^/dose on Days 1 and 8, Dacarbazine 200 mg/m^2^/dose on Days 1-5, Doxorubicin 30 mg/m^2^/dose on Days 6-7  Cycle 5: Cisplatin 40 mg/m^2^/dose on Days 1-4, Etoposide 100 mg/m^2^/dose on days 1-4, Vindesine 3 mg/m^2^/dose on Day 1  Cycle 6: Vincristine 1.5 mg/m^2^/dose on Days 1 and 8, Dacarbazine 200 mg/m^2^/dose on Days 1-5, Doxorubicin 30 mg/m^2^/dose on Days 6-7 |

Appendix S2: Search terms utilized for initial reference list.

**((((neuroblastoma and chemotherapy) OR (neuroblastoma and induction)) OR (neuroblastoma and phase)) OR (neuroblastoma and pilot)) OR (neuroblastoma and response)** Filters: **Clinical Trial, Clinical Trial, Phase I, Clinical Trial, Phase II, Clinical Trial, Phase III, Clinical Trial, Phase IV, Comparative Study, Controlled Clinical Trial, Multicenter Study, Randomized Controlled Trial, from 1995 – 2024**

((("neuroblastoma"[MeSH Terms] OR "neuroblastoma"[All Fields] OR "neuroblastomas"[All Fields]) AND ("chemotherapy s"[All Fields] OR "drug therapy"[MeSH Terms] OR ("drug"[All Fields] AND "therapy"[All Fields]) OR "drug therapy"[All Fields] OR "chemotherapies"[All Fields] OR "drug therapy"[MeSH Subheading] OR "chemotherapy"[All Fields])) OR (("neuroblastoma"[MeSH Terms] OR "neuroblastoma"[All Fields] OR "neuroblastomas"[All Fields]) AND ("inducted"[All Fields] OR "inducting"[All Fields] OR "induction"[All Fields] OR "inductions"[All Fields])) OR (("neuroblastoma"[MeSH Terms] OR "neuroblastoma"[All Fields] OR "neuroblastomas"[All Fields]) AND ("phase"[All Fields] OR "phase s"[All Fields] OR "phases"[All Fields])) OR (("neuroblastoma"[MeSH Terms] OR "neuroblastoma"[All Fields] OR "neuroblastomas"[All Fields]) AND ("pilot s"[All Fields] OR "piloted"[All Fields] OR "piloting"[All Fields] OR "pilots"[MeSH Terms] OR "pilots"[All Fields] OR "pilot"[All Fields])) OR (("neuroblastoma"[MeSH Terms] OR "neuroblastoma"[All Fields] OR "neuroblastomas"[All Fields]) AND ("response"[All Fields] OR "responses"[All Fields] OR "respon

sive"[All Fields] OR "responsiveness"[All Fields] OR "responsivenesses"[All Fields] OR "responsives"[All Fields] OR "responsivities"[All Fields] OR "responsivity"[All Fields]))) AND ((clinicaltrial[Filter] OR clinicaltrialphasei[Filter] OR clinicaltrialphaseii[Filter] OR clinicaltrialphaseiii[Filter] OR clinicaltrialphaseiv[Filter] OR comparativestudy[Filter] OR controlledclinicaltrial[Filter] OR multicenterstudy[Filter] OR randomizedcontrolledtrial[Filter]) AND (1995:2024[pdat]))

Appendix S3: Regimens from included studies with number of patients treated, study level end induction response rate (EIR), complete response rate (CR), and progressive disease (PD) rate.

| **Regimen** | **Publication** | **PMID** | **Number of patients treated** | **Primary outcome: End induction response (EIR) rate (%)** | **CR rate at end induction (%)** | **PD rate at end induction (%)** |
| --- | --- | --- | --- | --- | --- | --- |
| ANBL0532 without GCSF | Whittle 2020 | 32729196 | 13 | 100 | 50 |  |
| Chicago Pilot II (without antibody) | Kletzel 2002 | 11980999 | 18 | 100 | 80 |  |
| Chicago Pilot II (with ch14:18) | Kletzel 2002 | 11980999 | 7 | 100 | 50 |  |
| SMC NB-2004 | Sung 2013 | 22635247 | 50 | 96 | 49 | 2 |
| N7 (Phase A) | Cheung 2001 | 11464891 | 24 | 95.8 |  |  |
| Regimen A3 (with one cycle A1) | Kaneko 2002 | 12439032 | 88 | 95.5 | 71.6 | 2.3 |
| Chemoimmunotherapy with hu14.18K322A (NB2012) | Furman 2022 | 34871104 | 64 | 95.3 | 34 | 0 |
| ICE/CECaT | Donfrancesco 2004 | 15176712 | 17 | 93.8 | 0 | 0 |
| SMC NB-2009 | Lee 2017 | 28511709 | 54 | 92.6 | 53.7 | 3.7 |
| Chicago Pilot No. 1 | Cohn 1997 | 9337055 | 11 | 90.9 | 27.3 | 9.1 |
| NMTRC012 | Kraveka 2022 | 35355452 | 20 | 89.4 | 21.1 | 5.3 |
| N5/N6 | Berthold 2020 | 32067684 | 211 | 89.3 | 27 | 8 |
| POG9341 without window | Zage 2008 | 18704922 | 54 | 88.9 |  |  |
| N8+N5/N6 | Berthold 2020 | 32067684 | 211 | 88 | 29 | 10 |
| COG ANBL02P1 | Park 2011 | 22010014 | 31 | 86.7 | 23.3 | 3.3 |
| ATO + SMHPO-N-2012 NB | Li 2021 | 33858561 | 22 | 86.4 | 54.5 | 13.6 |
| EPiC | Pradhan 2006 | 16206215 | 25 | 85.7 | 9.5 | 14.2 |
| NB97 | Berthold 2005 | 16129365 | 295 | 84.7 |  |  |
| Rapid COJEC | Pearson 2008 | 18308250 | 130 | 84.1 | 44 | 2 |
| ThaiPOG-NB-13HR | Rujkijyanont 2019 | 31619207 | 107 | 82.2 | 1.9 | 0 |
| Regimen A1 | Kaneko 2002 | 12439032 | 22 | 81.8 | 40.9 | 13.6 |
| ANBL12P1 | Granger 2021 | 33823167 | 146 | 80 | 22.4 | 4.1 |
| COG ANBL0532 | Park 2019 | 31454045 | 652 | 79.7 |  | 7.1 |
| POG9340 + 9341 | Zage 2008 | 18704922 | 88 | 79.5 |  |  |
| COG A3973 | Kreissman 2013 | 23890779 | 486 | 78.6 | 22.4 | 14.7 |
| NB96 | Monnereau-Laborde 2011 | 21744481 | 20 | 76.9 |  |  |
| CCG 3891 | Matthay 1999 | 10519894 | 379 | 75.4 | 33.4 | 14.9 |
| MSKCC-N5 | Garaventa 2021 | 34152804 | 317 | 74.5 | 3 | 1 |
| OPEC/OJEC | Pearson 2008 | 18308250 | 132 | 73.4 | 38 | 9 |
| D-CECaT | Donfrancesco 1995 | 7576980 | 65 | 73.1 | 11.6 | 3.8 |
| rCOJEC without Filgrastim | Ladenstein 2010 | 20567002 | 120 | 72 |  |  |
| ANBL09P1 | Weiss 2021 | 34028986 | 53 | 71.7 | 24.5 | 18.9 |
| rCOJEC + Filgrastim | Ladenstein 2010 | 20567002 | 119 | 71 |  |  |
| rCOJEC | Garaventa 2021 | 34152804 | 313 | 70.3 | 3 | 3 |
| JN-H-11 | Yoneda 2024 | 38577760 | 64 | 67.2 | 3.1 | 3.1 |
| NB87 | Coze 1997 | 9396394 | 192 | 64.3 | 1 | 5 |

Appendix S4: Study level end induction response rate (EIR) as a predictor of 3-year EFS, 5-year EFS, 3-year OS, and 5-year OS using linear regression weighted by number of subjects

|  | **EIR** | |
| --- | --- | --- |
|  | Coefficient (95% CI) | p-value |
| EFS 3-yr | -0.063 [-1.241, 1.114] | 0.906 |
| EFS 5-yr | 1.093 [-0.191, 2.378] | 0.082 |
| OS 3-yr | -0.321 [-1.487, 0.846] | 0.526 |
| OS 5-yr | 0.315 [-0.854, 1.485] | 0.557 |

Appendix S5: Key toxicities of included regimens

| **Regimen** | **Publication (First Author, Year)** | **Number of patients treated (n)** | **Incidence of documented infections during induction regimen (%)** | **Completion rate of induction regimen (%)** | **Incidence of ototoxicity, any grade (%)** | **Toxic death rate (%)** | **Incidence of secondary malignancy (%)** |
| --- | --- | --- | --- | --- | --- | --- | --- |
| SMC NB-2004 | Sung 2013 | 50 |  |  | 76.2 | 0 |  |
| POG9340 + 9341 | Zage 2008 | 88 |  |  |  | 0 |  |
| POG9341 without window | Zage 2008 | 54 |  |  |  | 0 |  |
| NB97 | Berthold 2005 | 295 |  |  |  | 0.7 |  |
| ANBL09P1 | Weiss 2021 | 53 |  | 86.1 |  | 0 |  |
| COG A3973 | Kreissman 2013 | 486 |  | 76.5 | 36.6 | 3 |  |
| ANBL12P1 | Granger 2021 | 146 |  | 70 |  | 1.4 |  |
| JN-H-11 | Yoneda 2024 | 64 | 41.3 | 100 | 2.6 | 0 |  |
| ANBL0532 without GCSF | Whittle 2020 | 13 | 75 | 66.7 |  | 0 |  |
| ThaiPOG-NB-13HR | Rujkijyanont 2019 | 107 |  | 100 |  | 0 |  |
| EPiC | Pradhan 2006 | 25 |  | 88 |  | 4 |  |
| OPEC/OJEC | Pearson 2008 | 132 |  | 84 |  | 3.2 |  |
| Rapid COJEC | Pearson 2008 | 130 |  | 84 |  | 4.1 |  |
| COG ANBL02P1 | Park 2011 | 31 |  | 96.8 |  | 0 |  |
| COG ANBL0532 | Park 2019 | 652 |  |  |  | 1.1 |  |
| NB96 | Monnereau-Laborde 2011 | 20 | 65 | 85 |  | 0 |  |
| CCG 3891 | Matthay 1999 | 379 |  |  |  |  | 0.79 |
| ATO + SMHPO-N-2012 NB | Li 2021 | 22 | 100 | 100 |  | 0 |  |
| SMC NB-2009 | Lee 2017 | 54 |  |  | 59.3 | 0 | 1.9 |
| rCOJEC + Filgrastim | Ladenstein 2010 | 119 |  |  |  | 3.4 |  |
| rCOJEC without Filgrastim | Ladenstein 2010 | 120 |  |  |  | 0 |  |
| NMTRC012 | Kraveka 2022 | 20 |  | 95 |  | 0 |  |
| Chicago Pilot II (without antibody) | Kletzel 2002 | 18 |  | 100 |  | 0 |  |
| Chicago Pilot II (with ch14:18) | Kletzel 2002 | 7 |  | 71.4 |  | 0 |  |
| Regimen A1 | Kaneko 2002 | 22 |  |  |  |  |  |
| Regimen A3 (with one cycle A1) | Kaneko 2002 | 88 |  |  |  |  |  |
| rCOJEC | Garaventa 2021 | 313 | 25 | 94 |  | 1 |  |
| MSKCC-N5 | Garaventa 2021 | 317 | 35 | 94 |  | 1 |  |
| Chemoimmunotherapy with hu14.18K322A (NB2012) | Furman 2022 | 64 |  | 96.9 |  | 0 |  |
| ICE/CECaT | Donfrancesco 2004 | 17 |  |  |  | 0 |  |
| D-CECaT | Donfrancesco 1995 | 65 |  | 87.7 |  | 0 |  |
| NB87 | Coze 1997 | 192 | 32.8 | 95 |  | 2.6 |  |
| Chicago Pilot No. 1 | Cohn 1997 | 11 |  | 92 |  | 0 |  |
| N7 (Phase A) | Cheung 2001 | 24 |  | 100 |  | 0 |  |
| N5/N6 | Berthold 2020 | 211 |  |  |  | 1 |  |
| N8+N5/N6 | Berthold 2020 | 211 |  |  |  | 1 |  |
| **Number (%) of regimens reporting the toxicity of interest** | | | **7 (19.4)** | **22 (61.1)** | **4 (11.1)** | **33 (91.7)** | **2 (5.6)** |
